# Joint Arthroplasty in Patients With Left Ventricular Assist Devices

**DOI:** 10.1016/j.artd.2026.102005

**Published:** 2026-03-31

**Authors:** Audrey N. Kobayashi, Stephen C. Moye, Clay B. Beagles, Joshua B. Davis, Antonia F. Chen, Vivek M. Shah

**Affiliations:** Department of Orthopaedic Surgery, Brigham and Women’s Hospital, Harvard Medical School, Boston, MA, USA

**Keywords:** LVAD, Total joint arthroplasty, Left ventricular assist device, Postoperative outcomes

## Abstract

**Background:**

There have been limited case series describing joint arthroplasty (JA) in patients with left ventricular assist devices (LVADs). In the largest retrospective matched cohort study to date, we compared the postoperative complications of JA among patients with and without LVADs.

**Methods:**

A retrospective study from January 1, 2014, to December 31, 2023, was conducted comparing patients undergoing JA with LVADs to controls without LVADs matched for gender, age, body mass index, history of myocardial infarction, arthroplasty type, year of surgery, and Elixhauser Comorbidity Index in a 3:1 fashion. There were 13 JA procedures in the LVAD group and 39 in the control group. Postoperative complications of interest included emergency department visits within 90 days, any postoperative infection within 1 year, postoperative thrombosis within 1 year, need for transfusion within 1 year, Patient-Reported Outcomes Measurement Information System physical function scores, as well as 2-year survival from revision and 2-year mortality.

**Results:**

LVAD patients had similar rates of 90-day emergency department visits (7.7% vs 23.1%; *P =* .42), thrombosis (7.6% vs 2.6%; *P =* .44), transfusion (30.8% vs 23.1%; *P* = .71), and any infection (7.6% vs 23.1%; *P* = .41) as control patients without LVADs. The proportion of patients reporting improvement in Patient-Reported Outcomes Measurement Information System physical function scores was similar (83% LVAD group vs 72% control; *P* = 1.00). There was one reoperation (7.7%) in the LVAD group and 7 reoperations (17.9%) in the control group (*P* = .66). Kaplan–Meier analysis demonstrated similar 5-year survival from reoperation (*P* = .12) and mortality (*P* = .95) between the 2 groups.

**Conclusions:**

This is the largest series of patients undergoing JA after an LVAD procedure. JA patients with LVADs had similar 1-year postoperative complications to their matched counterparts.

## Introduction

Left ventricular assist devices (LVADs) are mechanical therapies used for the treatment of heart failure. They can be used to support patients as they undergo heart transplant evaluation, as they await transplantation, or as destination therapy. Based on the Society of Thoracic Surgeons Intermacs 2023 annual report, the volume of LVADs peaked in 2019, and more recently, approximately 2500 patients received an LVAD in 2022 [1]. Reported survival rates of LVAD patients are 64.6% at 3 years and 47.8% at 5 years [1], and therefore it is not surprising that LVAD patients will need to undergo noncardiac surgery. Upward of 49% of LVAD patients undergo noncardiac surgery for procedures such as endoscopy, general surgery, and orthopaedic surgery [2,3].

Limited reports have been published of joint arthroplasty (JA) procedures among LVAD patients [[4], [5], [6], [7]]. JA is an important set of procedures in the LVAD population, as these procedures have been shown to improve cardiac function and fitness, including outcomes such as oxygen consumption, maximum workload, and exercise duration [8,9]. Thus, not only will LVAD patients likely undergo JA procedures due to their increased survival rate, but JA can be an important component in helping them optimize cardiac function.

Despite these benefits, careful thought must be exercised when considering JA in LVAD patients. First, LVAD patients not only suffer from heart failure but also often have multiple concomitant medical comorbidities that make them higher risk surgical candidates. Second, LVAD patients have an increased risk of infection, most commonly due to driveline infections, though pump pocket infections and bloodstream infections have also been reported [10]. Any infection in the JA population is concerning, as periprosthetic joint infection (PJI) is a catastrophic complication. Third, LVAD patients have a higher risk for bleeding complications. The increased risk of bleeding among LVAD patients is due to the necessary use of anticoagulants, acquired von Willebrand factor deficiency, and platelet dysfunction [7,11]. Fourth, LVAD patients are at risk for thrombosis, in particular thrombotic stroke, with up to a 20% occurrence among this population [12]. Finally, the anesthetic plan must be made carefully as the use of general anesthesia and positioning the patient in the lateral position can affect both preload and systemic vascular resistance in LVAD patients with non-pulsatile flow [4]. The use of spinal anesthesia is a possibility; however, careful balance must be achieved to avoid spinal epidural hematoma or a thrombotic event while patient is off of anticoagulation [6]. All of the above factors make it imperative that there is close multispecialty collaboration in the management of LVAD patients undergoing JA.

Previous studies of JA among LVAD patients have been limited to case reports and small patient series [4,5,6,7] that have lacked a control group. This retrospective case-control study represents the largest cohort of LVAD patients undergoing JA, and postoperative complications were compared to a matched cohort of non-LVAD patients undergoing JA. We aimed to report on the perioperative complications as well as mid-term postoperative complications of this growing JA patient population. We hypothesized that 1-year postoperative complications would be similar between the 2 groups.

## Material and methods

### Study population

This retrospective study was approved by our institutional review board. All patients who underwent JA between January 1, 2014, and December 31, 2023, who had a previous history of LVAD placement at the study institutions were identified through our combined medical centers’ billing database. Patients who underwent total hip arthroplasty (THA), total knee arthroplasty (TKA), unicompartmental knee arthroplasty (UKA), and hip hemiarthroplasty were included. Similarly, a control group of patients was generated by querying our combined medical centers’ billing database for patients who underwent these same arthroplasty procedures. Preliminary data for matching were collected via chart review.

Patients were then propensity score matched with controls in a 3:1 fashion on the following variables: gender, age, body mass index, history of myocardial infarction, arthroplasty type, year of surgery, and Elixhauser Comorbidity Index. Given the inclusion of arthroplasty type as a variable in the matching model, patients could not be matched at the level of procedure type in an exact 3:1 ratio.

### Postoperative complications

Postoperative complications of interest included any postoperative infection, thrombosis, and transfusion occurring at any point during a 1-year follow-up period. Patients without documented complications 1 year after surgery were considered to be complication-free, regardless of the timing of documented orthopaedic surgery follow-up. Emergency department (ED) visits within 90 days were also recorded as well as perioperative change in patient-reported outcome metrics of Patient-Reported Outcomes Measurement Information System (PROMIS) physical function scores. Finally, we calculated the 2-year survival from all-cause joint revision as well as the overall 2-year survival.

### Statistical analysis

Comparisons were made between the LVAD and control groups. Categorical variables were compared using chi-square testing. Multiple groups were compared using Fisher’s exact test. Continuous variables were compared using Wilcoxon rank-sum testing. Survival analysis was performed using Kaplan–Meier estimates, and a log-rank test was used to draw inferences. Patients exited the survival analysis at the end of their documented orthopaedic follow-up or at the incidence of the event of interest. A *P* value less than 0.05 was considered significant. All analyses were performed with Stata 18.0 (StataCorp. 2023. Stata Statistical Software: Release 18. College Station, TX: StataCorp LLC).

## Results

There were 13 procedures across 8 patients in the LVAD group and 39 procedures in the control group in 37 patients. All patients in the LVAD group underwent the procedure as destination therapy. The median documented orthopaedic follow-up in the LVAD group was 1.81 years, ranging from 38 days to 9.85 years. Meanwhile, the control group median was 1.05 years, ranging from 6 days to 8.49 years. In the LVAD group, there were 7 TKAs, 3 UKAs, 2 THAs, and 1 hip hemiarthroplasty. In the control group, there were 25 TKAs, 4 UKAs, 9 THAs, and 1 hip hemiarthroplasty. The 2 groups were well-matched in preoperative characteristics (Table 1). The mean age was 74.3 years ±6.8 for the LVAD group and 71.5 years ±10.5 for the control group (*P* = .37). All patients in the LVAD group were white compared to 92.3% in the control group (*P* = .56). Both groups were entirely men. The mean body mass index for both groups was similar (27.4 kg/m^2^ ± 3.9 for the LVAD group and 25.4 kg/m^2^ ± 3.6 for the control group; *P* = .09). The mean Elixhauser Comorbidity Index was also similar between the 2 groups (52.2 ± 17.1 for the LVAD group and 49.3 ± 19.1 for the control group; *P* = .62). Both groups had a similar proportion of smokers and those with a history of myocardial infarction (Table 1).

**Table 1:** Patient characteristics.

Characteristic	LVAD group (n = 13)	Control group (n = 39)	*P* value
Operation type			
Total knee arthroplasty	7 (53.8%)	25 (64.1%)	.63
Unicompartmental knee arthroplasty	3 (23.1%)	4 (10.3%)	-
Total hip arthroplasty	2 (15.4%)	9 (23.1%)	-
Hemiarthroplasty	1 (7.7%)	1 (2.6%)	-
Men	13 (100%)	39 (100%)	1.00
Race			
White	13 (100%)	36 (92.3%)	.56
Non-white	0	3 (7.7%)	-
Mean age	74.3 ± 6.8	71.5 ± 10.5	.37
Mean BMI	27.4 ± 3.9	25.4 ± 3.6	.09
Mean Elixhauser Comorbidity Index	52.2 ± 17.1	49.3 ± 19.1	.62
Active smoker	9 (69.3%)	25 (64.1%)	1.00
History of myocardial infarction	10 (76.9%)	29 (74.4%)	1.00
Mean length of stay, surgical admission	6.2 days	4.6 days	.30

BMI, body mass index.

The need for perioperative transfusion at the time of JA was similar between the LVAD (30.8%) and controls groups (23.1%, *P* = .71, Table 2). The rate of postoperative thrombosis was also similar between the 2 groups (7.6% LVAD vs 2.6% non-LVAD; *P* = .44). The thrombosis case was related to pump thromboses in the LVAD group and the thrombosis event in the control group was related to a deep vein thrombosis. Finally, LVAD patients had similar postoperative infections compared to the control group (7.6% vs 23.1%; *P* = .41), as all postoperative infections among the LVAD group were driveline infections while PJI was the most common for the control group (44% of infections). The other infection types included pneumonia (22%), bloodstream infection (22%), and cellulitis (11%).

**Table 2:** Postoperative outcomes among LVAD and control groups.

Outcome	LVAD group (n = 13)	Control group (n = 39)	*P* value
Perioperative transfusion within 1 y	4 (30.8%)	9 (23.1%)	.71
Postoperative thrombosis within 1 y	1 (7.7%)	1 (2.6%)	.44
Postoperative infection within 1 y	1 (7.7%)	9 (23.1%)	.42
ED visit within 90 d	1 (7.7%)	9 (23.1%)	.42
Complete PROMIS recorded	9 (69.2%)	28 (71.8%)	-
Postop improvement	7 (77.8%)	20 (71.4%)	1.00
All-cause reoperation	1 (7.7%)	7 (17.9%)	.66

**Figure 1:**
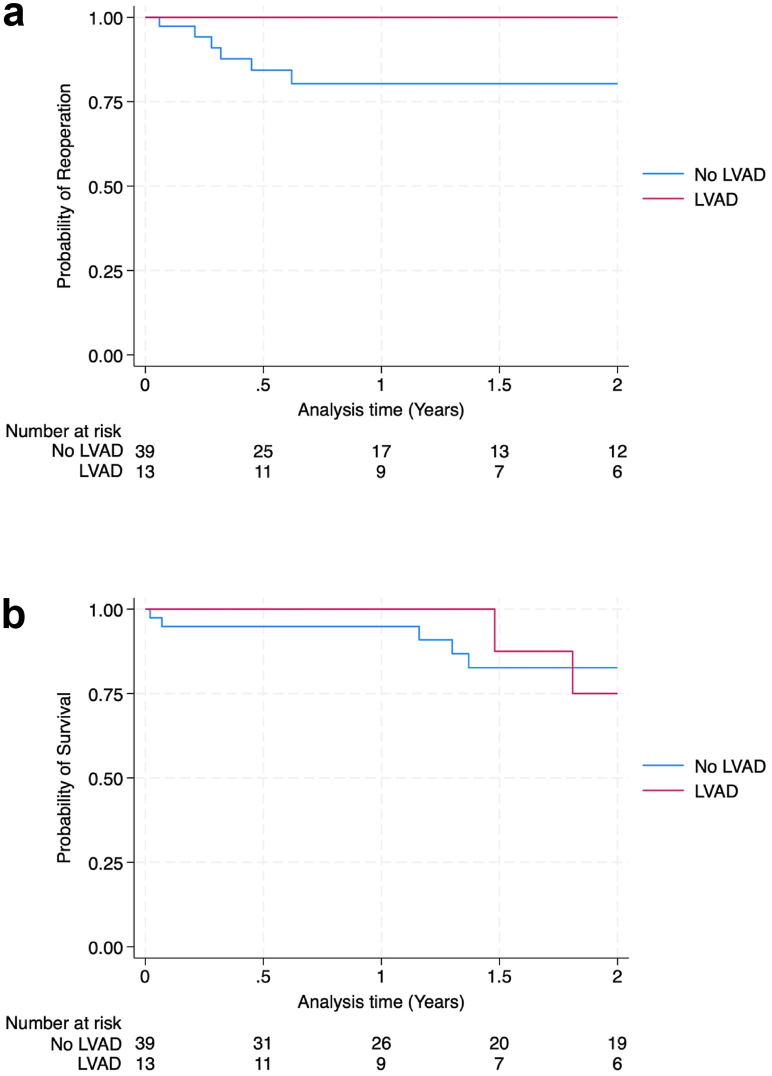
Kaplan–Meier analysis of 2-year survival from (a) all-cause reoperation and (b) mortality.

Other postoperative complications were also similar between the 2 groups. The proportion of patients who had an ED visit within 90 days of their operation was similar (7.7% LVAD vs 23.1% non-LVAD; *P* = .42). Nine of the LVAD patients (69.2%) had both preoperative and postoperative PROMIS physical function scores recorded, while 28 control patients (71.8%) had both scores recorded. Among the LVAD patients, 77.8% had an improvement in their postoperative scores and similarly, 71.4% had an improvement in the control group (*P* = 1.00). One patient in the LVAD group (7.7%) and 7 patients in the control group (17.9%) had an all-cause reoperation. Finally, we performed a Kaplan–Meier analysis to analyze both 2-year survival from reoperation (*P* = .12) as well as the overall survival (*P* = .95), and these were similar between the 8 groups (Fig. 1).

## Discussion

As outcomes improve, LVAD patients will continue to have a need for noncardiac surgeries. Among those procedures, JA is important given that it can restore functional status and directly improve cardiovascular function—both factors which are critical to LVAD patients. Despite this value, performing JA in LVAD patients can be high risk given their comorbidities. There has been a paucity of data on JA outcomes among LVAD patients, limited to case reports and small case series [4,5,6,7]. This study represents the largest cohort of LVAD patients undergoing JA compared to a matched control group of JA patients without LVADs.

The care of LVAD patients requiring JA requires careful and coordinated multidisciplinary care. The surgeon needs to have comfort operating on patients with multiple medical comorbidities, with requisite partnership from anesthesiology. Care of these patients at our institution involves regular involvement of specialty consults including cardiology, infectious disease, and general medicine. Patients require careful preoperative optimization of cardiac status with specific planning of perioperative anticoagulation management. Any sign of infection must be managed aggressively given the high prevalence of driveline infections among LVAD patients. Finally, general medicine input is critical to ensuring the optimal care of other myriad comorbidities the patient may have.

The results of this study suggest that LVAD patients undergoing JA have similar postoperative complications compared with matched JA patients without an LVAD. It is notable that the rates of perioperative complications at the 1 year mark including transfusion, infection, thrombosis, as well as all-cause reoperation, functional status, and mortality were similar between both groups. The 90-day ED visit rate in this study is similar to more general JA populations, which had an overall rate of 10.3% [13]. All postoperative infections in the LVAD patients were driveline infection, which is a commonly reported complication of LVADs [10]. Our observed rate of 7.6% among LVAD patients is comparable to literature for LVAD patients that report a 13.8% rate of driveline infections [14]. This increase in infections did not lead to a greater need for reoperation due to PJI and may indicate prompt treatment minimizing the likelihood of progression to PJI.

Although there was no difference between the groups, the revision rates and mortality rates observed in this study were higher than previously reported large studies of general JA patients [15]. The higher revision and mortality rates could be due to this study’s baseline sicker patient population with more comorbidities. This study was not designed to understand causation; however, we suspect that the observed similar postoperative complications between the groups such as thrombosis and perioperative blood loss can be attributed to the skilled multi-disciplinary care of LVAD patients.

It is worth emphasizing that a large majority of patients in the LVAD group reported an improvement in functional status as recorded by the PROMIS physical function scores. JA can play a critical role in maintaining good physical function among LVAD patients, which in turn, can be important in enhancing their quality of life [16].

There were multiple limitations to this study. One limitation of the present study was that it is a small retrospective cohort of patients which limits the generalizability of the study results. In addition, although the 2 groups in this study were well-matched in the variables that were selected, there could be additional important comorbidities that were not captured that could account for differences in these 2 groups other than the presence of LVAD. In addition, given that matching was performed on several pre-operative characteristics, it is possible that there may be asymmetry in the type of comorbidities present between the 2 groups. We sought to enhance our LVAD cohort by using a multicenter design, however, both institutions were from the same geographic area which may not apply to other geographic locations. Another limitation of this study is that we did not analyze the time between the placement of LVAD and JA procedure. There could be differences in outcomes based on the duration of LVAD use that we could not detect. We also cannot exclude the possibility that there are differences among different JA procedures since this was a pooled analysis of hip and knee arthroplasty procedures including both partial and total joint replacement. Subset analyses based on duration of LVAD and type of arthroplasty would be challenging given the small sample size within the subgroups. Finally, we did not stratify the LVAD cohort by their indication for LVAD, and it is possible that there are differences among subpopulations (eg, bridge to transplant vs destination therapy) that were not appreciated in this pooled analysis.

While the present study’s results were similar between the LVAD patients and those without an LVAD, the sample size was overall small and therefore likely not powered to detect small differences in postoperative outcomes. Our results suggest that JA may be of value to patients with an LVAD to help them improve physical function and quality of life.

## Conclusions

LVAD patients undergoing JA have similar postoperative complications as patients undergoing JA without LVAD, though the overall sample size was small.

## Conflicts of interest

A.F. Chen received royalties from Stryker; is a paid consultant for Adaptive Phage Therapeutics, Avanos, BICMD, Convatec, Ethicon, Heraeus, IrriMax, Osteal Therapeutics, Peptilogics, Pfizer, Smith & Nephew, Stryker, and TrialSpark; has stock or stock options in Hyalex, Irrimax, Osteal Therapeutics, Sonoran, and IlluminOss; received research support from Adaptive Phage Therapeutics, Elute, Peptilogics, and Sectra; received royalties, financial, or material support from Taylor & Francis Group, *Journal of Bone and Joint Surgery*, and UpToDate; is a member of the medical/orthopaedic publications editorial/governing board at *Journal of Arthroplasty, Journal of Bone and Joint Infection, Journal of Bone and Joint Surgery*, and *Arthroplasty Today*; and is a board member/committee appointments for AJRR and AAHKS; all other authors declare no potential conflicts of interest.

For full disclosure statements refer to https://doi.org/10.1016/j.artd.2026.102005.

## CRediT authorship contribution statement

**Audrey N. Kobayashi:** Conceptualization, Data curation, Formal analysis, Investigation, Methodology, Writing – original draft, Writing – review & editing. **Stephen C. Moye:** Conceptualization, Data curation, Formal analysis, Investigation, Methodology, Writing – review & editing. **Clay B. Beagles:** Data curation, Formal analysis, Investigation, Methodology, Writing – review & editing. **Joshua B. Davis:** Data curation, Formal analysis, Methodology, Writing – review & editing. **Antonia F. Chen:** Conceptualization, Investigation, Methodology, Resources, Supervision, Writing – review & editing. **Vivek M. Shah:** Investigation, Resources, Supervision, Writing – review & editing.

## Appendix A Supplementary data

Conflict of Interest Statement for Kobayashi

Conflict of Interest Statement for Chen

Conflict of Interest Statement for Beagles

Conflict of Interest Statement for Davis

Conflict of Interest Statement for Moye

Conflict of Interest Statement for Shah
