# Supplementary material for: Joint Arthroplasty in Patients With Left Ventricular Assist Devices
Source: Arthroplast Today. 2026 Mar 31;39:102005. doi: 10.1016/j.artd.2026.102005 (PMC13068593; doi:10.1016/j.artd.2026.102005)
Supplement: Conflict of Interest Statement for Chen [file mmc2.docx]

# INDIVIDUAL CONFLICT OF INTEREST STATEMENT

***The Journal of Arthroplasty***

(Adopted from the American Academy of Orthopaedic Surgeons disclosure statement)

The following form **must be filled out completely and submitted by each author (example, 6 authors, 6 forms). If no discloser is required, please write/type “none” at the end of each sentence.**

**Manuscript Title Joint Arthroplasty in Patients with a Left Ventricular Assist Devices (LVADs)**

1. Royalties from a company or supplier (The following conflicts were disclosed)

Stryker

2. Speakers bureau/paid presentations for a company or supplier (The following conflicts were disclosed)

None

3A. Paid employee for a company or supplier (The following conflicts were disclosed)

None

3B. Paid consultant for a company or supplier (The following conflicts were disclosed)

Adaptive Phage Therapeutics, Avanos, BICMD, Convatec, Ethicon, Heraeus, IrriMax, Osteal Therapeutics, Peptilogics, Pfizer, Smith and Nephew, Stryker, TrialSpark

3C. Unpaid consultants for a company or supplier (The following conflicts were disclosed)

None

4. Stock or stock options in a company or supplier (The following conflicts were disclosed)

Hyalex, Irrimax, Osteal Therapeutics, Sonoran, IlluminOss

5. Research support from a company or supplier as a Principal Investigator (The following conflicts were disclosed)

Adaptive Phage Therapeutics, Elute, Peptilogics, Sectra

6. Other financial or material support from a company or supplier (The following conflicts were disclosed)

None

7. Royalties, financial or material support from publishers (The following conflicts were disclosed)

Taylor & Francis Group, Journal of Bone and Joint Surgery, UpToDate

8. Medical/Orthopaedic publications editorial/governing board (The following conflicts were disclosed)

Journal of Arthroplasty; Journal of Bone and Joint Infection; Journal of Bone and Joint Surgery; Arthroplasty Today

9. Board member/committee appointments for a society (The following conflicts were disclosed)

AJRR, AAHKS

**Each author must sign AND print or type his/her name, date and submit a separate form**

In addition, one BLINDED Conflict of Interest form (no author names used) should be submitted per manuscript with all author disclosures.


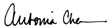


Antonia F. Chen, MD/MBA 7/1/2024

Author Name (Print or Type) Author Signature Date
